# Elucidation of the Differences in Cinobufotalin’s Pharmacokinetics Between Normal and Diethylnitrosamine-Injured Rats: The Role of P-Glycoprotein

**DOI:** 10.3389/fphar.2019.00521

**Published:** 2019-05-17

**Authors:** Xiaojing Zhang, Tong Liu, Yidan Zhang, Fanye Liu, Haiying Li, Dong Fang, Chaojie Wang, Hua Sun, Songqiang Xie

**Affiliations:** ^1^School of Pharmacy, Institute for Innovative Drug Design and Evaluation, Henan University, Kaifeng, China; ^2^The Key Laboratory of Natural Medicine and Immuno-Engineering, Henan University, Kaifeng, China; ^3^School of Pharmacy, Institute of Chemical Biology, Henan University, Kaifeng, China

**Keywords:** hepatocellular carcinoma, cinobufotalin, UPLC-MS/MS, pharmacokinetics, P-glycoprotein

## Abstract

Cinobufotalin is one of the major anti-tumor components isolated from toad venom and has been used in the clinical therapy of hepatocellular carcinoma (HCC), known as Cinobufacini injection. However, the pharmacokinetic (PK) behaviors of cinobufotalin *in vivo* with HCC are still unknown. Hence, we have established a HCC model in Sprague Dawley (SD) rats induced by diethylnitrosamine (DEN), named as DEN-injured rats. Then, we developed and validated a sensitive and rapid ultra-performance liquid chromatography-tandem mass spectrometric (UPLC-MS/MS) method to quantify cinobufotalin in rat plasma. This UPLC-MS/MS method was successfully used to characterize the PK behaviors of cinobufotalin in normal and DEN-injured rats after intravenous (i.v.) injection at a dosage of 2.5 mg/kg. Cinobufotalin pharmacokinetics was well described by the two-compartment pharmacokinetic model and the PK parameters were calculated using WinNonlin 3.3 software. The transfer rate constant of cinobufotalin from the central compartment to the peripheral compartment (k_12_) in DEN-injured rats was significantly greater than that in normal rats (*p* < 0.01), accompanied by the shorter half-life for the distribution phase (t_1/2α_). Additionally, the elimination rate constant (K_10_) and clearance (CL) values in DEN-injured rats were significantly higher than that in normal rats (*p* < 0.05 for K_10_ and *p* < 0.001 for CL, respectively). Therefore, the values of areas under concentration – time curve (AUC) and the liver concentration of cinobufotalin in DEN-injured rats was obviously lower than that in normal rats (*p* < 0.001 and *p* < 0.01, respectively). This indicated that the PK behaviors of cinobufotalin will be altered in rats with HCC. In addition, P-glycoprotein (P-gp) has shown higher expression in live tissues of DEN-injured rats. Furthermore, cinobufotalin was identified as the substrate of P-gp using MDCK II and MDCK-MDR1 cell models for the first time. Consequently, P-gp will play an important role in the disposition of cinobufotalin *in vivo*, which provided a new combination therapy for the clinical treatment of HCC.

## Introduction

Hepatocellular carcinoma (HCC) is one of the most common primary malignancy of the liver and the second mortality cancer in the worldwide (Ferlay et al., 2013; Raoul et al., 2018). Various factors can lead to development of HCC, such as infection of hepatitis B virus or hepatitis C virus (Forner et al., 2018), non-alcoholic fatty liver disease (Estes et al., 2018), metabolic syndrome (Jinjuvadia et al., 2014), diabetes (Schlesinger et al., 2013), and obesity (Marrero et al., 2005). In most cases, HCC develops from non-alcoholic steatohepatitis (Charlton, 2018), which lead to the late diagnose and reduced the possibility of successful surgical resection or liver transplantation during the treatment (Kansagara et al., 2014). In addition, patients are prone to acquired drug resistance during the long time exposed to chemotherapeutic agents (Niu et al., 2017), such as sorafenib, the first systemic drug approved by the US Food and Drug Administration for advanced HCC (Raoul et al., 2018). Therefore, the treatment of HCC remains a challenge and the new effective antineoplastic agents for HCC are still needed (Cidon, 2017).

**FIGURE 1:**
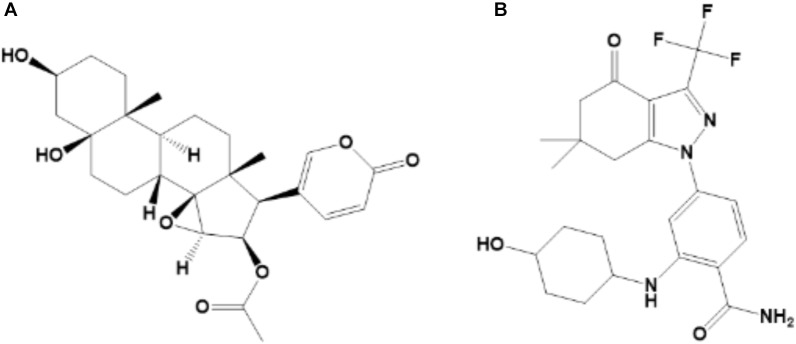
Chemical structure of cinobufotalin **(A)** and SNX-2112 **(B)**.

Traditional Chinese Medicine (TMC) is widely used due to high efficiency and low toxicity in the treatment of various diseases. Toad venom (also known as Chan’su) is derived from the dried skin secretions of giant toads (Bufo gargarizans Cantor or Bufo melanostictus Suhneider) and is currently used in clinical practice, such as Cinobufacini injection, to treat HCC (Kamano et al., 2002; Tang et al., 2008; Hu et al., 2011; Zhang et al., 2018). Cinobufotalin (Figure 1A), a bufadienolide in chemical structure, is one of the major anti-tumor components of Cinobufacini injection (Tao et al., 2010; Zhang et al., 2018). The octanol-water partition coefficient (LogP) of cinobufotalin calculated using Advanced Chemistry Development (ACD/Labs) Software V11.02 was 0.174 ± 0.614, which indicated that the lipophilicity of this compound was slightly better than its water-solubility. Meanwhile, cinobufotalin has shown proper physico-chemical properties, such as the molecular weight was 458.55 (<500), the LogP and water solubility (117.43 mg/L, measured in our laboratory) at pH 7.0 was within the optimal range (LogP ≤ 3, solubility > 50 mg/L), the polar surface area was 106 Å^2^ (≤120 Å^2^) and the number of rotatable bonds was 5 ( ≤ 7) (Teague et al., 1999; Sirois et al., 2005; Vistoli et al., 2008). This implied that cinobufotalin has shown excellent druggability.

Cinobufotalin has shown anti-HCC potential through inhibiting cell growth and inducing cell apoptosis, and has been used in the clinical therapy for advanced HCC patients (Chen et al., 2008; Tao et al., 2010; Guo et al., 2018). In addition, our research results indicated that cinobufotalin also could prevent the metastasis of HCC cells (HepG2 and Huh-7 cells) (Supplementary Figures S1, S2) through inhibit the epithelial-mesenchymal transition progress (date not yet published). However, no reports have been published regarding the pharmacokinetic behavior of cinobufotalin in rats with HCC, so the identification of the optimum administration routes and the evaluation of subsequent biological effects, remain big challenges. Therefore, it is necessary to develop a validated and sensitive analytical method that can be used to monitor cinobufotalin *in vivo*.

P-glycoprotein (P-gp), encoded by *MDR1* gene, widely exists in various tissues (Sugawara, 1990) and mediates the transmembrane transport of endobiotics and xenobiotics using energy from the ATP hydrolysis (Robey et al., 2018). Numerous studies have certified the higher expression level of P-gp in tumors/cancer cells, such as liver tumors, lung adenocarcinoma and colon cancer cells (Bai et al., 2017; Wu M. et al., 2018; Yan et al., 2018). Moreover, overexpress of P-gp is one of the main precipitating factors for the emergence of multi-drug resistance through influencing the absorption, distribution, metabolism and excretion of drugs during chemotherapy (Robey et al., 2018). Throughout recent years, many researches have been conducted to found or design new P-gp inhibitors, which can be used in combination therapy for cancers to improve the bioavailability and efficacy of therapeutic compounds or drugs (Ma et al., 2018; Riganti et al., 2018). Hence, it is imperative to define the role of P-gp in cinobufotalin transport, and the results are expected to provide a reference for future clinical research and rational application of cinobufotalin.

The diethylnitrosamine (DEN)-injured animal model could imitate the pathogenesis of HCC and was often used to explore the pharmacodynamics and possible mechanisms of new active agents (Fuchs et al., 2014; Orrù et al., 2018; Wen et al., 2018). In this study, we have developed a sensitive and reliable UPLC-MS/MS method which can be used to quantify cinobufotalin in plasma samples *in vivo*. We also investigated and compared the differences in PK behaviors of cinobufotalin between normal and DEN-injured rats. Furthermore, the role of P-gp involvement into cinobufotalin transport was identified. This might provide a reference for the rational use of cinobufotalin in future preclinical and clinical research.

## Materials and Methods

### Chemicals and Reagents

Cinobufotalin (purity > 98%, Figure 1A) was purchased from Baoji Herbest Bio-Tech Co., Ltd. (Baoji, China). SNX-2112 (purity > 98%; Figure 1B) was purchased from MedChemexpress (Monmouth Junction, NJ, United States) and used as the internal standard (IS). Carprofen injection was purchased from National Animal Health Products Research Center of China. PEG400, Ketamine, Xylazine, Acetopromazine, ethanol, dimethyl sulphoxide (DMSO) and Sodium chloride solution (0.9%) were analytical grade and purchased from Sigma-Aldrich (St Louis, MO, United States). Acetonitrile, methanol and water, which were LC-MS grade, were bought from Merk KGaA (Darmstadt, Germany).

### Instruments and Conditions

The anti-HCC effect of cinobufotalin *in vivo* requires further study. Before that, it is necessary to characterize the PK behavior of cinobufotalin *in vivo*. UPLC-QTOF/MS was employed to determine the amount of cinobufotalin in rat plasma after intravenous (i.v.) injection. Chromatographic separation was performed using Waters ACQUITY UPLC system (Milford, MA) and BEH column (2.1 × 50 mM, 1.7 mm; Waters). The temperature of the column was set at 40°C and the automatic sample chamber was kept at 4°C. The sample were eluted using a gradient of formic acid (0.1%) in water (mobile phase A) vs. formic acid (0.1%) in acetonitrile (mobile phase B) at a flow rate of 0.45 mL/min. The gradient elution condition was 10% B at 0–1.0 min, 10–40% B at 2.0–3.0 min, 40–50% B at 3.0–5.0 min, 50–90% B at 5.0–6.0 min, and 90–10% B at 6.0–7.0 min.

Data were collected in the positive mode on the Xevo G2 QTOF/MS (Waters) using the electrospray ionization source (ESI) in full scan mode (50–1200 Da). The capillary, sampling cone and extraction cone voltages were 3000, 40, and 4V, respectively. The desolvation gas (nitrogen) and cone gas was set to 800 and 30 L/h, respectively. The desolvation and source temperature were 350 and 100°C, respectively. In order to ensure the mass accuracy, an external reference (lock spray) of 2 ng/mL leucine enkephalin (m/z 556.2771) was infused at a rate of 5 μL/min during the sample run.

### Preparation of Rat Plasma Samples

Standard stock solutions of cinobufotalin and IS were all prepared in DMSO at the concentration of 10 and 2 mg/mL, respectively. Stock solutions were stored at -20°C until they were used for preparing working solutions after dilution with a certain volume of acetonitrile. The rat plasma (blank or containing cinobufotalin and IS) was treated with protein precipitation. In briefly, 10 μL of cinobufotalin working solution was added into 90 μL of rat plasma and mixed by vortex for 10 s. Five hundred microliter of the IS working solution (20 ng/mL) was added to the rat plasma and mixed by vortex for 60 s. Then the mixture was centrifuged at 12,000 g for 15 min, and the supernatant was transferred to a clean tube and dried using Eppendorf Concentrator Plus (Hamburg, Germany). The dried residues were re-dissolved in 100 μL of acetonitrile/water (50:50, v/v). After centrifugation (12,000 g, 15 min), the supernatant was subjected to UPLC-QTOF/MS for analyses.

### Method Validation

For method validation, the selectivity, linearity, limit of quantification (LOQ), limit of detection (LOD), intra-day and inter-day viability, extraction recoveries, matrix effects and stability were evaluated using blank rat plasma as described in our previous published papers (Lin et al., 2017).

#### Selectivity

A selective method should ensure that the analytes and the IS can be identified from other substances present in matrix (blank rat plasma). Rat plasma containing conbufotalin and IS was prepared as described in *Preparation of rat plasma samples* section. Blank matrix was detected simultaneously as a control.

#### Linearity

Standard curve samples containing different concentrations of cinobufotalin (12.5–5000 ng/mL) in rat plasma were prepared by half dilution method.

#### LOQ and LOD

The LOQ and LOD were determined by the signal-to-noise ratio of 10:1 and 3:1, respectively.

#### Intra-Day and Inter-Day Viability

Six replicates of quality control (QC) samples at three concentration levels (20, 200, and 2000 ng/mL) in rat plasma were tested in the same day and in three different days to determine the intra-day and inter-day precisions and accuracies. Precision was expressed as the relative standard deviation (RSD) of the QC sample and accuracy was expressed as a percentage of the measured value to the true value (%).

#### Extraction Recoveries and Matrix Effects

Three concentration levels (20, 200, and 2000 ng/mL) of cinobufotalin in rat plasma were prepared. Extraction recoveries and matrix effects were calculated using (Equations 1 and 2).

(1) $$Extraction\;recovery=\frac{peak\;area\;of\;analyte\;spiked\;blank\;plasma\;before\;extraction}{peak\;area\;of\;analyte\;spiked\;blank\;plasma\;after\;extration}\times 100\%$$

(2) $$Matrix\;effect=\frac{peak\;area\;of\;analyte\;spiked\;blank\;plasma}{peak\;area\;of\;analyte\;spiked\;aqueous\;solution}\times 100\%$$

#### Stability

In addition, the stabilities of cinobufotalin at 20, 200, and 2000 ng/mL in different storage conditions were evaluated, including short term stability (such as 25°C for 4, 8, 12, and 24 h and 4°C for 4, 8, 12, and 24 h), long term stability (-80°C for 10 days) and three freeze-thaw cycles in rat plasma. Stabilities were expressed as the concentrations after different operations to the concentration at time zero. All of the samples were processed as described in *Preparation of rat plasma samples section*. A 5 μL aliquot was injected to UPLC-QTOF/MS for analyses.

### Pharmacokinetic Studies

#### Animals

Male Sprague Dawley rats (200–220 g), used for pharmacokinetic (PK) studies, were purchased from Beijing Vital River Laboratory Animal Technology Co., Ltd. (Beijing, China). All animals were housed under standardized condition (12 h light/dark schedule), and they were kept 1 week prior to experiments for acclimation. All animal experiments were carried out in accordance with the National Institutes of Health guide for the care and use of Laboratory animals (NIH Publications No. 8023, revised 1978) and were approved by the Medical and Research Ethics Committee of College of Medicine, Henan University.

#### Experimental Design

For PK studies, the rats were randomly divided into two experimental groups (10 rats for normal rats group and 20 rats for DEN-injured rats group, considering the animal casualties during the modeling process). HCC rat model was established by intraperitoneal administration of 50 mg/kg DEN two times per week (Fuchs et al., 2014), and the control group rats were treated with normal saline in the same way. After 14 weeks of administration, three rats were randomly selected and anesthetized by anesthesia cocktail (Ketamine 50 mg/mL, Xylazine 3.3 mg/mL and Acetopromazine 3.3 mg/mL). Then the liver was taken out and photographed to determine whether the animal model was successfully constructed.

Before PK experiment, a cannula was introduced into the jugular vein of the anesthetized rat and administrated carprofen subcutaneously after the surgery. After observation for 24 h, when the activity and excretion of rats were normal, rats were dosed with cinobufotalin at 2.5 mg/kg through jugular vein cannula. Blood samples (200 μL) were withdrawn at each pointed time (5, 10, 15, 30, 45 min, and 1, 1.5, 2, 4, 6, 8, 12, and 24 h) after dosing, and whole blood was transferred into tubes pre-treated with heparin (1000 U/mL). Then an equal volume of saline was injected into rats through jugular vein cannula immediately. The blood samples were immediately centrifuged at 4°C (8000 g, 10 min) to obtain the plasma, and the plasma samples were stored at -80°C until analysis. After sampling at the last time point, all rats were anesthetized immediately. Blood was washed out with ice-cold saline through the hepatic portal vein, and then liver tissues were removed, weighed and stored at -80°C until analysis.

#### Samples Preparation

Hundred microliter of plasma was transferred to a new clean tube, and samples were treated with protein precipitation assay as described in *Preparation of rat plasma samples section*. Then calibration curve was prepared to quantify the amount of cinobufotalin in plasma samples according to the steps as described in *method validation part.*

The liver tissues were homogenized with saline solution (1/2, v/w). 200 μL of tissue homogenate was mixed with 1 mL of IS working solution (20 ng/mL) and vortexed for 1 min. After centrifuged at 12,000 × *g* for 15 min (4°C), the supernatant was collected and dried using Eppendorf Concentrator Plus (Hamburg, Germany). The dried residues were re-dissolved in 200 μL of acetonitrile/water (50:50, v/v). After centrifugation (12,000 g, 15 min), the supernatant was subjected to UPLC-QTOF/MS for analyses.

#### Pharmacokinetic Analysis

**FIGURE 2:**
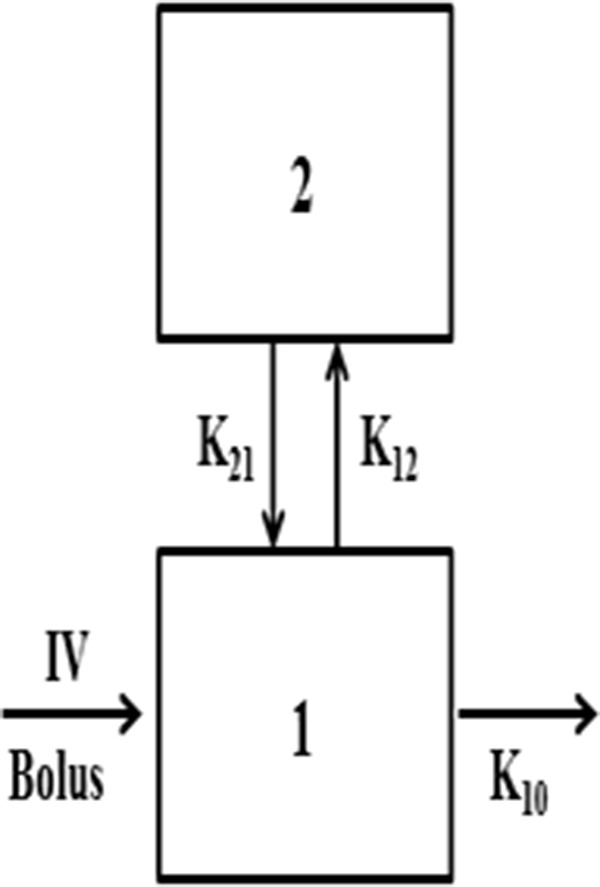
Two-compartment IV-Bolus PK model, with first-rate elimination constant (K_10_), and distribution rates K_12_ and K_21_.

PK and statistical data analyses were performed using WinNonlin 3.3. The two-compartment model (two-compartment i.v.-Bolus, macro-constants, no lag time, first order elimination, Figure 2) was selected to obtain the PK parameters of cinobufotalin in rats. The model equation was as followed:

(3) C(t)=A∗EXP(−Alpha∗t)+B∗EXP(−Beta∗t)

### Cinobufotalin Cellular Uptake Studies

Madin Darby canine kidney (MDCK II) cells stably transfected with multidrug resistance 1 gene (MDCK-MDR1 cells) were conducted and used in cinobufotalin uptake studies. Wild type MDCK II or MDCK-MDR1 cells were seeded into six-well plates at the destiny of 2 × 10^5^ cells/well and maintained at 37°C under 5% CO_2_ in DMEM with 10% FBS. The well-known P-gp inhibitor, verapamil (100 μM), was used as the positive control to evaluate the effect of P-gp on cinobufotalin uptake (Bicker et al., 2017).

The uptake experiment was performed when the cells reached 100% confluency. First, cells were washed three times using 37°C Hanks’ balanced salt solution (HBSS) buffer (pH = 7.2). Then, 2 mL HBSS (37°C, pH = 7.2) buffer containing cinobufotalin (5 and 20 μM) were added and incubated with cells in the absence or presence of potential P-gp inhibitors, verapamil (100 μM). After incubation for 2 h, the incubation medium was removed, and cells were washed three times using ice-cold HBSS buffer. Then 400 μL of ice-cold MeOH:H_2_O (1:1, v/v) were added into each well, the cells were collected and sonicated for 15 min in ice-cold water. The mixture was centrifuged at 4°C (12,000 g, 10 min), and the supernatant was subjected to UPLC-QTOF/MS for analyses.

### Bidirectional Transport Experiments

MDCK II and MDCK-MDR1 cells were seeded into 12-well Transwell polycarbonate membrane inserts (12 mm diameter inserts, 0.4 μm pore size, Corning Costar) at a density of 6 × 10^5^ cells/well. Cells were cultured at 37°C under 5% CO_2_ in DMEM with 10% FBS, and the culture medium was refreshed every other day. Experiments were conducted when the tight junctional cell monolayer formed. First, the cells were washed three times using 37°C preheated HBSS buffer (pH = 7.4) and preincubated in a shaking incubator for 30 min at 37°C. Then the transport studies were initiated after the blank HBSS buffer and dosing solution (HBSS buffer containing cinobufotalin at a final concentration of 20 μM) were added to the receiver compartment and donor compartment, respectively. Verapamil, if used, was added to both compartment during the incubated period. The volumes of the apical side (AP) and basolateral side (BL) were 0.5 and 1.5 mL, respectively. Samples (100 μL) were taken from the receiver compartment every 30 min during the incubation period of 120 min, and then replaced with the same volume of dosing solution (containing cinobufotalin). At the end of the incubated period, aliquots of 100 μL were withdrawn from the donor compartment to calculated mass balance. All samples were mixed with 50 μL acetonitrile and centrifuged at 4°C (12,000 g, 10 min. The supernatant was subjected to UPLC-QTOF/MS for analyses.

The apparent permeability (cm/s) from apical-to-basal (P_app,AP-BL_) and basal-to-apical (P_app,BL-AP_) were calculated according to the following Equation (4), where *dQ/dt* is the rate of drug permeation (nmol/s), *A* is the area of polycarbonate membrane (*A* = 1.12 cm^2^), and C_0_ is the initial concentration of cinobufotalin in the donor compartment.

(4) $$P_{app}=\frac{\left(dQ/dt\right)}{A\times C_{0}}$$

The efflux ratio (ER) and net flux ratio were calculated according to Equations (5) and (6) as described in our previous research (Sun et al., 2017). If the net flux ratio value ≥ 2, then it indicated that the test compound was the substrate of P-gp.

(5) $$ER=\frac{P_{app,\;\;BL-AP}}{P_{app,\;\;AP-BL}}$$

(6) $$Net\;flux\;ratio=\frac{ER_{MDCK-MDRI}}{ER_{MDCK\;II}}$$

### Western Blotting Analysis

Western blotting analysis was performed as described previous (Liu T. et al., 2018). In brief, protein samples (40 μg) were analyzed by sodium dodecyl sulfatepolyacrylamide gel electrophoresis (10% acrylamide gels) and transferred to polyvinylidene fluoride membranes (0.22 μM, Millipore, Bedford, MA, United States). After blocking for 1 h, the membranes were incubated with primary antibodies (1:1000 dilution for P- gp and β-actin antibody) at 4°C overnight, followed by anti-rabbit or anti-mouse horseradish peroxidaseconjugated secondary antibody. Target protein bands were visualized with enhanced chemiluminescence and imaged by autoradiography. And then the protein bands were scanned in grayscale and subjected to semi-quantitative analysis using Quantity One software.

### Statistical Analysis

Data were presented as mean ± standard deviation (SD). Statistically significant differences between two group data were analyzed by two-tailed Student’s *t*-test. The level of significance was set at *p* < 0.05.

## Results

### Chromatography and Mass Spectrometry Analysis for Cinobufotalin in Rat Plasma

**FIGURE 3:**
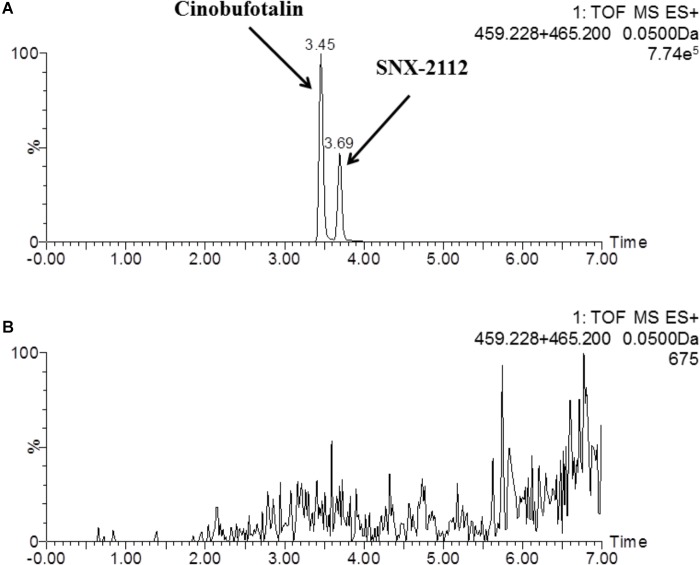
Representative mass chromatograms of spiked cinobufotalin and SNX-2112 (IS) in rat plasma **(A)**, and blank rat plasma **(B)**.

**FIGURE 4:**
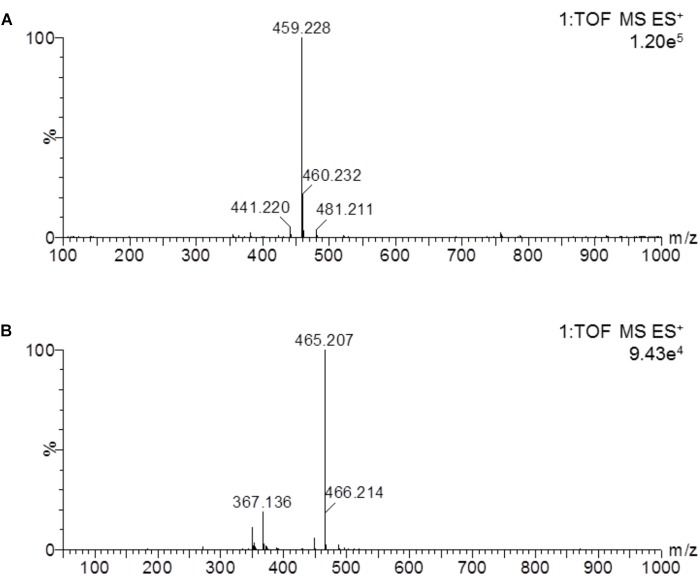
Mass spectrum of cinobufotalin and SNX-2112 in a prepared rat plasma sample. The results showed the molecular ion 459.228 [M+H]^+^ of cinobufotalin **(A)** and 465.207 [M+H]^+^ for SNX-2112 **(B)**.

In this study, a rapid and sensitive UPLC-MS/MS method was developed for the quantification of cinobufotalin in rat plasma. The developed method was simple, using protein precipitation assay with acetonitrile. SNX-2112 was chosen as the internal standard (IS) because of its chemical structure and property similarity to cinobufotalin. In addition, SNX-2112 showed high sensitivity in positive ion mode (Lin et al., 2017), which was consistent with cinobufotalin (Figure 3). After injection to UPLC-MS/MS system, cinobufotalin in plasma sample was observed at 3.45 min with the [M+H]^+^ ion at 459.228 (Figure 3, 4), and IS was observed at 3.69 min with the [M+H]^+^ ion at 465.207 (Figure 3, 4).

### Method Validation

#### Specificity

As shown in Figure 3, the peaks of cinobufotalin and IS were well separated in rat plasma samples. In addition, there was no interference from other substances. The analysis method was considered to be specific for evaluating the quality of cinobufotalin and IS in rat plasma.

#### Linearity, LOQ, and LOD

In the range of 12.5–5000 ng/mL, cinobufotalin showed good linearity (y = 0.545x + 2.251) in rat plasma, with the correlation coefficient (R^2^) of > 0.999. The LOQ and LOD of cinobufotalin was 5 and 2 ng/mL, respectively.

#### Intra-Day and Inter-Day Precision and Accuracy

The Intra-day and inter-day viabilities were determined for three different concentrations of cinobufotalin on the same day or on three different days. The data are shown in Table 1. The intra-day relative standard deviation (RSD) were 5.4, 2.5, and 2.2% at 20, 200, and 2000 ng/mL, and the inter-day relative standard deviation (RSD) were 4.0, 2.1, and 0.7% at 20, 200, and 2000 ng/mL, respectively. The intra-day accuracies were 99.0, 101.2, and 103.3% at 20, 200, and 2000 ng/mL, and inter-day accuracies were 100.3, 96.8, and 104.3% at 20, 200, and 2000 ng/mL, respectively. The assay for cinobufotalin was validated with an acceptable intra-day and inter-day precision and accuracy within 10%.

**Table 1:** Intra-day and inter-day precisions and accuracy of cinobufotalin at low, medium, and high concentrations in rat plasma.

Concentration (ng/mL)	Intra-day (*n* = 6)		Inter-day (*n* = 6)	
		
	Precisions (RSD, %)	Accuracy (%)	Precisions (RSD, %)	Accuracy (%)
20	5.4	99.0	4.0	100.3
200	2.5	101.2	2.1	96.8
2000	2.2	103.3	0.7	104.3


#### Extraction Recoveries and Matrix Effects

Plasma samples were prepared by precipitation with acetonitrile. As shown in Table 2, the extraction recoveries and the matrix effects of cinobufotalin at three different concentrations (20, 200, and 2000 ng/mL) were not less than 90 and 95%, respectively.

**Table 2:** Extraction recoveries and matrix effects of cinobufotalin at low, medium, and high concentrations in rat plasma.

Concentration (ng/mL)	Extraction recovery (%)		Matrix effect (%)	
		
	Average (%)	RSD (%)	Average (%)	RSD (%)
20	99.0	1.9	89.5	1.6
200	94.8	2.2	93.0	3.2
2000	102.3	2.4	92.8	5.3


#### Stability

As shown in Table 3, the recoveries of cinobufotalin in plasma at -80°C for 10 days and after three freeze-thaw cycles were between 95.6 and 100.9%, which demonstrated that the cinobufotalin in plasma samples was stable under storage conditions. In addition, we also evaluated the stability of cinobufotalin in 50% accetonitrile solution at 4°C and room temperature (25°C) after storage for 4, 8, 12, and 24 h. The results showed that cinobufotalin was stable in 50% accetonitrile solution with the recoveries between 95.5 and 103.7% (Table 3), thus demonstrated that cinobufotalin was stable in the process of sample preparation and analysis.

**Table 3:** Stabilities of cinobufotalin at low, medium, and high concentrations in rat plasma under different storage conditions.

Concentration (ng/mL)	In rat plasma (%)		In 50% Acetonitrile solution (%)							
		
	-80°C for 10 days	Freezing-thaw3 cycles	4°C for different time				25°C for different time			
				
			4 h	8 h	12 h	24 h	4 h	8 h	12 h	24 h
20	99.9 ± 3. 7	99.2 ± 0.8	97.5 ± 5.7	101.9 ± 3.6	99.1 ± 2.5	100.4 ± 2.7	99.5 ± 4.4	97.1 ± 2.3	97.7 ± 4.6	95.5 ± 4.3
200	100.9 ± 1.2	95.6 ± 1.6	102.6 ± 6.8	99.5 ± 4.0	97.4 ± 2.9	96.7 ± 2.6	100.1 ± 2.8	101.2 ± 2.0	99.5 ± 4.3	99.0 ± 2.8
2000	97.3 ± 5.6	99.2 ± 0.6	102.0 ± 5.0	103.7 ± 3.5	96.3 ± 4.0	97.9 ± 2.5	100.3 ± 3.0	99.1 ± 1.1	98.4 ± 3.5	100.5 ± 3.3


*Data are presented as mean ± SD (n = 3).*

In summary, the acceptable results obtained in method validation, include good linearity, precision, accuracy, recovery and stability, indicated that the sample preparation with protein precipitation and analysis using UPLC-MS/MS system was suitable for cinobufotalin quantification in rat plasma.

### UPLC-MS/MS Method Application to PK Studies in Rats

**FIGURE 5:**
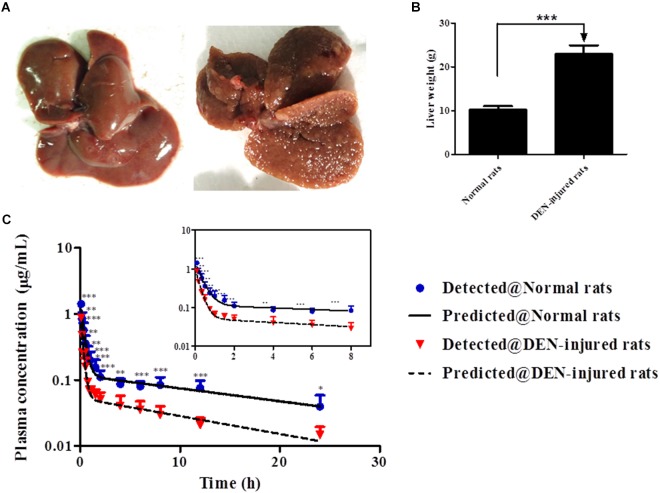
**(A)** After treated with normal saline or DEN for 14 weeks, the liver tissues were collected. **(B)** The liver weight of normal and DEN-injured rats. **(C)** Mean plasma concentrations in each time point of cinobufotalin after i.v. injection (2.5 mg/mL) in normal rats and DEN-injured rats, and the internal panel plot represented the data of first 8 h. Each point is shown as the average concentration and standard deviations (*n* = 6). ^∗^*p* < 0.05, ^∗∗^*p* < 0.01, and ^∗∗∗^*p* < 0.001.

Compared to normal liver, rough surface with multinodular were observed in the liver of DEN-injured rats (Figure 5A). And meanwhile, the liver weight of DEN-injured rats was significantly higher than that in normal rats (Figure 5B, *p* < 0.001). Furthermore, the validated analytical method was successfully used to identify the PK behaviors of cinobufotalin in rats. The mean plasma concentration-time profiles after i.v. bolus of cinobufotalin in the two groups of rats were presented in Figure 5C and consisted of two phases on the semilog scale, which was more obviously in the internal panel (the experimental data for the first 8 h) of Figure 5C. An initial distribution phase characterized by rapid decline in drug concentration, followed by a terminal elimination phase with slower rate of decline in drug concentration. This was a typical plasma concentration-time profile after i.v. bolus administration of drugs that follow two-compartment pharmacokinetic model. And besides, the two-compartment model fitted the data very well as the detected data was very close to the predicted data. Moreover, the Akaike information criterion (AIC) values of the model were the lowest compared to other models, such as non-compartment model, one-compartment model and three-compartment model, which indicated two-compartment model was most appropriate model for the data fitting.

The PK parameters of cinobufotalin were calculated by the two-compartmental method using WinNonlin 3.3, and the results were shown in Table 4. After i.v. administration, there were no significant differences in the initial concentrations (C_0_), the large distribution volumes (V) and the elimination half-lives (t_1/2β_) of cinobufotalin between the two groups of rats. However, the transfer rate constant of cinobufotalin from the central compartment to the peripheral compartment (k_12_) in normal rats was obviously lower than that in DEN-injured rats (1.8 ± 0.5 1/h vs. 3.2 ± 0.8 1/h, *p* < 0.01), and consequently, the half-life for the distribution phase (t_1/2α_) was longer (0.3 ± 0.1 h vs. 0.2 ± 0.1 h, *p* < 0.01). Meanwhile, the AUC values of cinobufotalin in normal rats was significantly higher than that in DEN-injured rats (2.6 ± 0.5 μg ⋅ h/mL vs. 1.1 ± 0.3 μg.h/mL, *p* < 0.001). In addition, the elimination rate constant (K_10_) and CL value of cinobufotalin in normal rats (1.0 ± 0.2 L/kg/h) was obviously lower (*p* < 0.05) than that in DEN-injured rats (2.4 ± 0.6 L/kg/h). These results indicated that cinobufotalin was eliminated rapidly in blood circulation in the HCC conditions and the retention time in blood was shortened.

**Table 4:** PK parameters of cinobufotalin in normal and DEN-injured rats after i.v. administration (2.5 mg/kg).

Parameters	Units	Normal rats	DEN-injured rats
K_10_	1/h	0.5 ± 0.2	1.0 ± 0.3^*^
K_12_	1/h	1.8 ± 0.8	3.2 ± 0.8^**^
K_21_	1/h	0.3 ± 0	0.3 ± 0.1
t_1/2α_	h	0.3 ± 0.1	0.2 ± 0.1^*^
t_1/2β_	h	13.2 ± 1.3	13.4 ± 5.8
C_0_	μg/mL	1.4 ± 0.4	1.1 ± 0.3
V	L/kg	2.1 ± 0.6	2.4 ± 0.6
CL	L/kg/h	1.0 ± 0.2	2.4 ± 0.6^***^
V_2_	L/kg	13.1 ± 1.3	31.5 ± 10.4^**^
CL_2_	L/kg/h	3.3 ± 0.4	8.1 ± 1.7^***^
AUC	μg^∗^h/mL	2.6 ± 0.5	1.1 ± 0.3^***^


*Data are presented as mean ± SD (n = 6). ^∗^p < 0.05, ^∗∗^p < 0.01, and ^∗∗∗^p < 0.001 compared with normal rats.*

### Liver Distribution of Cinobufotalin

**FIGURE 6:**
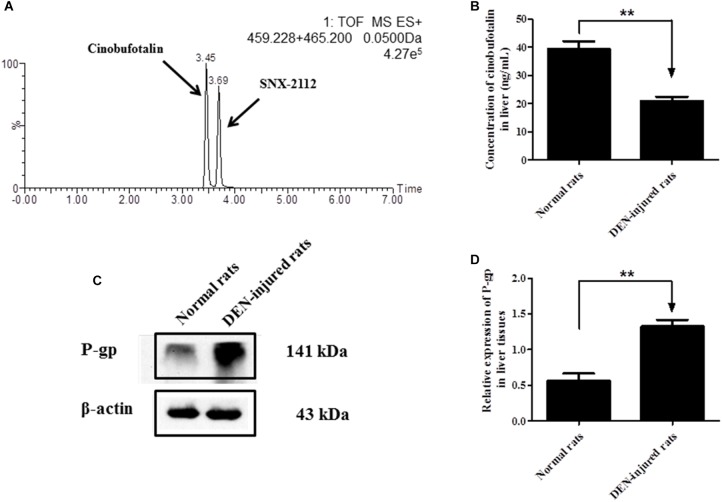
**(A)** Representative mass chromatograms of spiked cinobufotalin and SNX-2112 (IS) in rat liver. **(B)** Concentration of cinobufotalin in liver tissues of normal and DEN-injured rats. **(C)** Expression of P-gp in liver tissues of normal and DEN-injured rats. **(D)** Semi-quantitative analysis of p-gp protein expression using Quantity One software. Each data point was the average of three determinations with the error bar representing the S.D. (*n* = 3). ^∗∗^*p* < 0.01.

The liver distribution of cinobufotalin was detected using UPLC-MS/MS method (Figure 6A). As shown in Figure 6B, the concentration of cinobufotalin in normal rats was significantly higher than that in DEN-injured rats (39.3 ± 4.9 vs. 21.0 ± 2.7 ng/mL, *p* < 0.01). Expectedly, the protein expression level of P-gp in DEN-injured rats was dramatically higher (*p* < 0.01) than that in normal rats by 136% (Figure 6C,D). These results enabled us to identify if P-gp played an important role in the transport of cinobufotalin. Then the intracellular accumulation and bidirectional transport studies for cinobufotalin using MDCK II and MDCK-MDR1 cells were conducted.

### Intracellular Accumulation of Cinobufotalin in MDCK II and MDCK-MDR1 Cells

**FIGURE 7:**
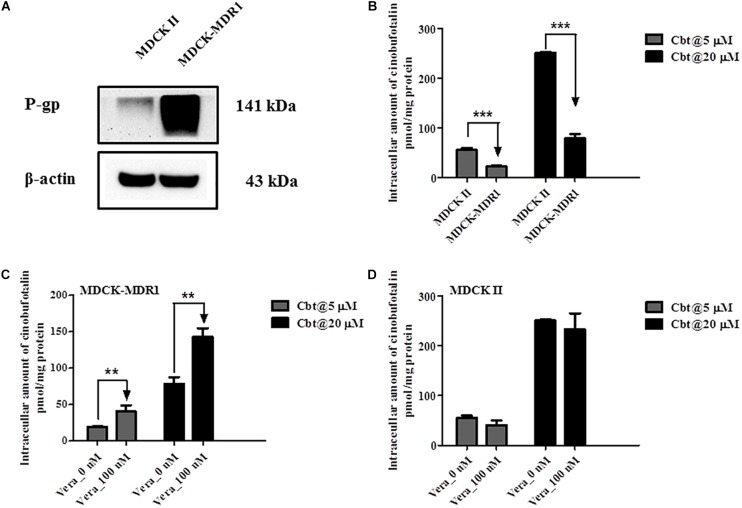
**(A)** Expression of P-gp in MDCK II and MDCK-MDR1 cells. **(B)** Intracellular accumulation of cinobufotalin in MDCK II and MDCK-MDR1 cells. **(C)** Effect of verapamil (100 μM) on the intracellular accumulation of cinobufotalin in MDCK-MDR1 cells. **(D)** Effect of verapamil (100 μM) on the intracellular accumulation of cinobufotalin in MDCK II cells. Each data point was the average of three determinations with the error bar representing the S.D. (*n* = 3). ^∗∗^*p* < 0.01 and ^∗∗∗^*p* < 0.001.

Western blotting results showed that P-gp was successfully overexpressed in MDCK-MDR1 cells compared to MDKC II cells (Figure 7A). At both high (20 μM) and low (5 μM) incubation concentration, the intracellular amount of cinobufotalin in MDCK II cells was obviously higher (*p* < 0.001) than that in MDCK-MDR1 cells (Figure 7B). However, the addition of verapamil (100 μM) reversed this tendency and resulted in markedly increased (*p* < 0.01) accumulation of cinobufotalin in MDCK-MDR1 cells at the dose of 5 and 20 μM (by 4.0 and 3.5-fold, respectively) (Figure 7C). By contrast, verapamil did not alter the accumulation of cinobufotalin in MDCK II cells (*p* > 0.05, Figure 7D).

### Transport of Cinobufotalin Across MDCK II and MDCK-MDR1 Cell Monolayers

Cinobufotalin showed directional transport in MDCK-MDR1 cells, as the P_app_ values of cinobufotalin from apical-to-basal (P_app,AP-BL_) and basal-to-apical (P_app,BL-AP_) were 33.82 ± 1.05 and 35.9 ± 1.3 cm/s (Table 5), respectively. Then the calculated ER values were 2.2 (> 2), which indicated that the transport of cinobufotalin was mediated by P-gp. The net flux ratio value of cinobufotalin calculated by Equation (6) was 2.1 (Table 5). When verapamil was used, the net flux ratio decreased by 50.7%. The net flux ratio higher than 2 combined with a reduction more than 50% in the presence of inhibitor (verapamil) proved that cinobufotalin was the substrate of P-gp.

**Table 5:** P_app_, ER, and net flux ratio of cinobufotalin in MDCK II and MDCK-MDR1 cell monolayers.

	MDCK II			MDCK-MDR1			
			
	P_app,AP-BL_ ( × 10^-6^cm/s)	P_app,BL-AP_ ( × 10^-6^cm/s)	ER	P_app,AP-BL_ ( × 10^-6^cm/s)	P_app,BL-AP_ ( × 10^-6^cm/s)	ER	Net flux ratio
Cinobufotalin	33.8 ± 1.1	35.9 ± 1.3	1.1	19.4 ± 1.3	42.4 ± 1.5^∗∗∗^	2.2	2.1
Cinobufotalin (with verapamil)	23.4 ± 2.2	24.8 ± 2.7	1.1	32.1 ± 1.5	34.5 ± 2.3	1.1	1.0


*Data are presented as mean ± SD (n = 3). ^∗∗∗^p < 0.001 compared with P_*app, AP-BL*_.*

## Discussion

In recent years, more and more TCM has been approved for the treatment of tumors due to their excellent pharmacological effects, such as anti-proliferation, anti-metastasis, pro-apoptosis, improving immunity and minimizing side effects of chemotherapy drugs. Cinobufotalin was one of the main active components of Toad venom (an important TCM), and was used alone or in combination for the treatment of HCC (Chen et al., 2008; Tao et al., 2010; Guo et al., 2018). However, many studies have focused on the evaluation of pharmacological activities of cinobufotalin, and few focused on the disposition of cinobufotalin *in vivo*, especially in disease model. In order to provide good guidance for optimal cinobufotalin administration conditions (e.g., dosage selection, dose interval) in future studies for the treatment of HCC, it is necessary to define the PK characters of cinobufotalin *in vivo*. Before that, a satisfactory analytical method is indispensable.

In this study, we have developed and validated a rapid, sensitive and specific UPLC-MS/MS analysis method for the quantification of cinobufotalin in rats. Then the validated method was successfully used to characterize the PK behaviors of cinobufotalin in normal and DEN-injured rats. Furthermore, the differences of PK behaviors of cinobufotalin between normal and DEN-injured rats were investigated. Although an analytical method using solid-phase extraction-high performance liquid chromatography (SPE-HPLC) for cinobufotalin in plasma has been reported (Liang et al., 2008), but this method needed more injection volume (20μL) and longer analysis time (30 min). Compared with that, the advantages of our method relied on its small injection sample volume (5μL), rapid analysis (7 min), simple plasma sample preparation procedure, satisfactory recoveries (>90%) and lower LOQ (5 ng/mL) and LOD (2 ng/mL), thus providing a much needed comprehensive method for the analysis of cinobufotalin in *in vivo* studies.

The PK behavior of cinobufotalin in rats after a single i.v. bolus conformed to two-compartmental model, and the concentration-time profile during the terminal elimination phase was linear on the semilog scale since it declined depending on the rate of cinobufotalin elimination. The plasma concentrations of cinobufotalin in the DEN-injured rats were significantly lower than those in normal rats at each time point, thus resulting in lower AUC in DEN-injured rats (Figure 5C). The calculated PK parameters have also changed accordingly (Table 4). The change in k_12_ affected the distribution rate and tissue distribution of cinobufotalin, such as, the larger k_12_ of cinobufotalin in DEN-injured rats resulted in faster distribution rate (shorter t_1/2α_) and higher amount of cinobufotalin distributing to the peripheral compartment (larger distribution volume, V_2_) (Hedaya, 2012). The larger CL of cinobufotalin indicated that the elimination rate of cinobufotalin in DEN-injured rats was faster, which was mainly attributed to the obviously lager k_10_ and k_12_ in DEN-injured rats. Meanwhile, the k_21_ value was slightly higher, although there is no significant difference. These significant differences suggested that HCC conditions have effect on the *in vivo* behavior of cinobufotalin.

Previous studies have demonstrated that Cytochrome P450 3A4 (CYP3A4), the most abundant P450 isoform expressed in human liver, played a predominant role in hydroxylation and deacetylation of bufodienolides, such as bufotalin, arenobufagin, and cinobufagin (Ma et al., 2011; Feng et al., 2017; Dai et al., 2019). Although CYP3A4 content is highest in the intestine as in the liver (Paine et al., 2006), but the liver remains the principal organ for bufodienolides metabolism mediated by CYP450 enzymes. That is because the total protein amount of CYP450 enzymes in the liver is 20–300 times that in the intestine (Paine et al., 1997; Yang et al., 2004; Gröer et al., 2014). Cinobufotalin belongs to bufodienolides and undergo hydroxylation and dehydrogenation in human liver microsomes, especially mediated by CYP3A4 (Han et al., 2016). Hence, we speculate that hepatic CYP450-mediated metabolism represents the major means by which the body eliminates cinobufotalin. Nevertheless, the disposition or metabolic behaviors of cinobufotalin *in vivo* have not been investigated at present. Furthermore, the hydroxylated metabolites have been considered to be the primary excretion form of bufodienolides after administration, which were mostly found in plasma and bile after administration (Ning et al., 2010; Wei et al., 2019). Accordingly we presume that cinobufotalin may experience extensively hydroxylation in the liver, and biliary excretion played a leading role in eliminating cinobufotalin metabolites.

Most HCC patients are advanced form chronic liver disease, showing liver function injure. It’s well known that diseases, such as liver injury, renal failure, inflammation and diabetes, could affect the expression or active of metabolism enzymes and/or transporters that in turn alter the pharmacokinetic (PK) behaviors of drugs *in vivo* (Jamwal et al., 2018; Varkhede et al., 2018). In addition, it has been reported that hydroxylation and dehydrogenation mediated by Cytochrome P450 (CYP450) enzymes, especially CYP3A4, were the major metabolic pathways of cinobufotalin *in vitro* and *in vivo* (Han et al., 2016). Hence the activity reduction of multiple CYP450 isoenzymes in the HCC rat models (Peng and Ding, 2015; Wu R. et al., 2018) should lead to slower clearance rate of cinobufotalin, showing lower k_10_. Because that k_10_ is the elimination rate constant that depends on metabolism and excretion of the compounds or drugs. However, the k_10_ and CL of cinobufotalin in DEN-injured rats was higher than that in normal rats. Meanwhile, the AUC of cinobufotalin in DEN-injured rats was lower. Furthermore, the concentration of cinobufotalin in liver tissues was also lower in DEN-injured rats. So we hypothesized that the efflux transporters, especially P-gp, may involvement in the disposition of cinobufotalin *in vivo*.

MDCK II and Caco-2 cells can differentiate structurally and functionally into polarized monolayers, referred as the apical side (AP) and basolateral side (BL) (Volpe, 2008). The wild type or transfected subclones of MDCK II and Caco-2 cells have been widely used to study permeability, transepithelial transport and efflux liabilities mediated by the main efflux transporters, such as P-gp, breast cancer resistance protein (BCRP) and multidrug resistance-associated proteins (MRPs) (Volpe, 2008; Liu L. et al., 2018; Xu et al., 2018). However, the formation of MDCK II cell monolayers needed shorter culture time than Caco-2 cells (Volpe, 2008). In addition, previous studies have reported that MDCK II has a good correlation with the Caco-2 cell model in evaluating active drug transport and permeation experiments (Volpe, 2011; Jin et al., 2014). Hence, we have used MDCK II and MDCK-MDR1 (overexpression of P-gp protein) to evaluated the effect of P-gp on cinobufotalin transport.

We have evaluated the protein expression level of P-gp in liver tissues using western blotting method. Not surprisingly, the expression level of P-gp in the liver of DEN-injured rats was significantly higher (by 136%, *p* < 0.01) than that in normal rats. Then we employed MDCK II and MDCK-MDR1 cell models to estimate the role of P-gp involvement into cinobufotalin transport. The efflux ratio (ER) value was higher than 2.0 in MDCK-MDR1 cell monolayers, indicated that P-gp mediate the active transport of cinobufotalin (Bicker et al., 2017). Furthermore, cinobufotalin was, for the first time, identified as the substrate of P-gp with the net flux ratio was higher than 2.0 (Bicker et al., 2017; Liu L. et al., 2018). After preincubation with verapamil, the noticeably reduction of net flux ratio ascertain cinobufotalin was the substrate of P-gp. These results indicated that P-gp may play an important role in the liver distribution and PK difference of cinobufotalin in normal and DEN-injured rats.

Since our current *in vivo* study provides evidence that HCC conditions affect the disposition of cinobufotalin, we only evaluated the concentration of cinobufotalin in plasma and liver tissues. The distribution of cinobufotalin in other important tissues, such as heart, spleen, lung, kidney, intestine and brain, should be evaluated as well. In addition, we anticipate that P-gp inhibitors may increase the exposure of cinobufotalin. However, further studies are still warranted to evaluate if the antitumor efficacy of cinobufotalin will be enhanced in combination with P-gp inhibitors. As previously reported, over dose of cinobufotalin could resulted in cardiotoxicity and neurotoxicity (Bick et al., 2002; Bi et al., 2016). Hence extensive attention should be attracted with the toxicity of cinobufotalin during the treatment of HCC patients in the future studies, especially in the present of P-gp inhibitors.

## Conclusion

In conclusion, we have developed a satisfactory analytical method for the detection of cinobufotalin in rats. The exposure of cinobufotalin in both plasma and liver greatly decreased in DEN-injured rats. Furthermore, cinobufotalin has been identified as the substrate of P-gp using MDCK-MDR1 cell models. Our results may have important clinical applications and P-gp inhibitors should be combined to enhance the plasma and liver exposure of cinobufotalin in the treatment of HCC.

## Ethics Statement

All animal experiments were carried out in accordance with the National Institutes of Health guide for the care and use of Laboratory animals (NIH Publications No. 8023, revised 1978) and were approved by the Medical and Research Ethics Committee of College of Medicine, Henan University.

## Author Contributions

XZ and HS participated in research design. XZ, TL, YZ, FL, HL, and HS conducted the experiments. DF, SX, and CW contributed new reagents or analytic tools. XZ, TL, YZ, and HS performed data analysis. XZ and HS wrote or contributed to the writing of the manuscript.

## Conflict of Interest Statement

The authors declare that the research was conducted in the absence of any commercial or financial relationships that could be construed as a potential conflict of interest.

## Abbreviations

AUC: area under concentration – time curve
DEN: diethylnitrosamine
ER: efflux ratio
HCC: hepatocellular carcinoma
i.v.: intravenous
IS: internal standard
MDCK: Madin-Darby canine kidney
P_app_: apparent permeability coefficient
P-gp: P-glycoprotein
PK: pharmacokinetic
UPLC-MS/MS: ultra-performance liquid chromatography-tandem mass spectrometry
