# Supplementary material for: Elucidation of the Differences in Cinobufotalin’s Pharmacokinetics Between Normal and Diethylnitrosamine-Injured Rats: The Role of P-Glycoprotein
Source: Front Pharmacol. 2019 May 17;10:521. doi: 10.3389/fphar.2019.00521 (PMC6533572; doi:10.3389/fphar.2019.00521)

# Development and validation of an ultra-performance liquid chromatography-tandem mass spectrometry method for the pharmacokinetics determination of cinobufotalin in diethylnitrosamine-injured rats

Xiaojing Zhang^a,1^, Tong Liu^a,1^, Yidan Zhang^a^, Fanye Liu^a^, Haiying Li^a^, Dong Fang^a^, Chaojie Wang ^b^, Hua Sun^a,^*, Songqiang Xie^c,^*

^a^Institute for innovative drug design and evaluation, School of Pharmacy, Henan University, N. Jinming Ave., Kaifeng, Henan 475004, China.

^b^The Key Laboratory of Natural Medicine and Immuno-Engineering, Henan University, N. Jinming Ave., Kaifeng, Henan 475004, China.

^c^Institute of Chemical Biology, School of Pharmacy, Henan University, N. Jinming Ave., Kaifeng, Henan 475004, China.

^1^These authors contribute equally to this work

* correspondence:

Hua Sun, Ph. D.

Email: sunhua_rain@hotmail.com

Songqiang Xie, Ph. D.

Email: xiesq@henu.edu.cn.

Supplementary material

# 1. Supplementary Experimental Procedures

**1.1 Cell lines and cell culture**

Hepatocellular cancer cells (HepG2 and Huh-7) were purchased from the cell bank of the Chinese Academy of Science (Shanghai, China). These cells were cultured in Dulbecco’s modified Eagle’s medium (DMEM) containing 10% fetal bovine serum (FBS), penicillin (100 units/mL) and streptomycin (100 units/mL) in a humidified atmosphere with 5% CO_2_ at 37 °C.

**1.2 Wound healing assays**

Wound healing assay was used to investigate the effect of cinobufotalin on the migration of HepG2 and Huh-7 cells. Cells were seeded into 6-well plates at the density of 4 × 10^5^ cells/well and maintained until reaching monolayer confluence. The cells were scratched using 20-μL pipette tips and rinsed with PBS buffer for three times. And then the cells were cultured in serum-free medium containing different concentrations of cinobufotalin (40, 60, 80 nM). After treatment with cinobufotalin for different lengths of time (0 and 24 h), the width of wound was measured and the images of cells were acquired using inverted microscope (Leica, Wetzlar, German). The rate of wound healing = [(the wound width of 0 h - 24 h)/0 h wound width] × 100%. All experiments were performed in triplicate.

**1.3 Cell viability assays**

Furthermore, the effect of cinobufotalin at 40, 60, 80 nM on hepatocellular cancer cells (HepG2 and Huh-7 cells) viability was conducted using Cell Counting Kit - 8 (CCK-8) assay (Sigma-Aldrich, St Louis, MO). In brief, cells were seeded into 96-well plates at 5 × 10^3^ cells/well and cultured for 24 h in DMEM medium as described in 1.1 section. Then various concentrations of cinobufotalin (40, 60, 80 nM) were added into cells. After 24 h treatment, 10 μL of CCK-8 solution was added into every well of the plate. The plate was incubated in the incubator for other 4 h, and then the absorbance was measured at 450 nm using a microplate reader (Thermo Scientific, Rockford, IL). The cell viability was calculated by the formula: cell viability (100%) = (OD value of the treated wells - OD value of the blank control wells) / (OD value of the negative control wells - OD value of the blank control wells).

# 2. Supplementary Experimental Results and Discussion

**2.1 Cinobufotalin inhibited the migration of HepG2 and Huh-7 cells**

The effects of cinobufotalin at different concentrations (40 nM, 60 nM and 80 nM) on cell migration were evaluated. The results indicated that the migration of HepG2 and Huh-7 cells was significantly attenuated by cinobufotalin in a dose-dependent manner (Fig.S1 and Fig.S2). Of note, cinobufotalin inhibited less than 15% of both HepG2 and Huh-7 cells growth at the doses of 40 nM, 60 nM and 80 nM, respectively (Fig. S1 C and Fig. S2 C). These results suggested that cinobufotalin inhibited the viability and migration of HepG2 and Huh-7 cells in a dose-dependent manner.

**Figure S1 Cinobufotalin attenuate the migration of HepG2 cells.** (A) The migratory properties of HepG2 cells were determined using wound healing assays (100× magnification). (B) The migration rate was calculated according to the wound width. (C) The inhibition effect of cinobufotalin ( 40, 60 and 80 nM) on the viability of HepG2 cells was evaluated using CCK-8 assays. Each bar is the average of the average of three determinations with the error bar representing the S.D. (n = 3). ***p < 0.001 compared with vehicle control.

**Figure S2 Cinobufotalin attenuate the migration of Huh-7 cells.** (A) The migratory properties of Huh-7 cells were determined using wound healing assays (100× magnification). (B) The migration rate was calculated according to the wound width. (C) The inhibition effect of cinobufotalin ( 40, 60 and 80 nM) on the viability of Huh-7 cells was evaluated using CCK-8 assays. Each bar is the average of the average of three determinations with the error bar representing the S.D. (n = 3). *p < 0.05, **p < 0.01 and ***p < 0.001 compared with vehicle control.

**Figure S1**


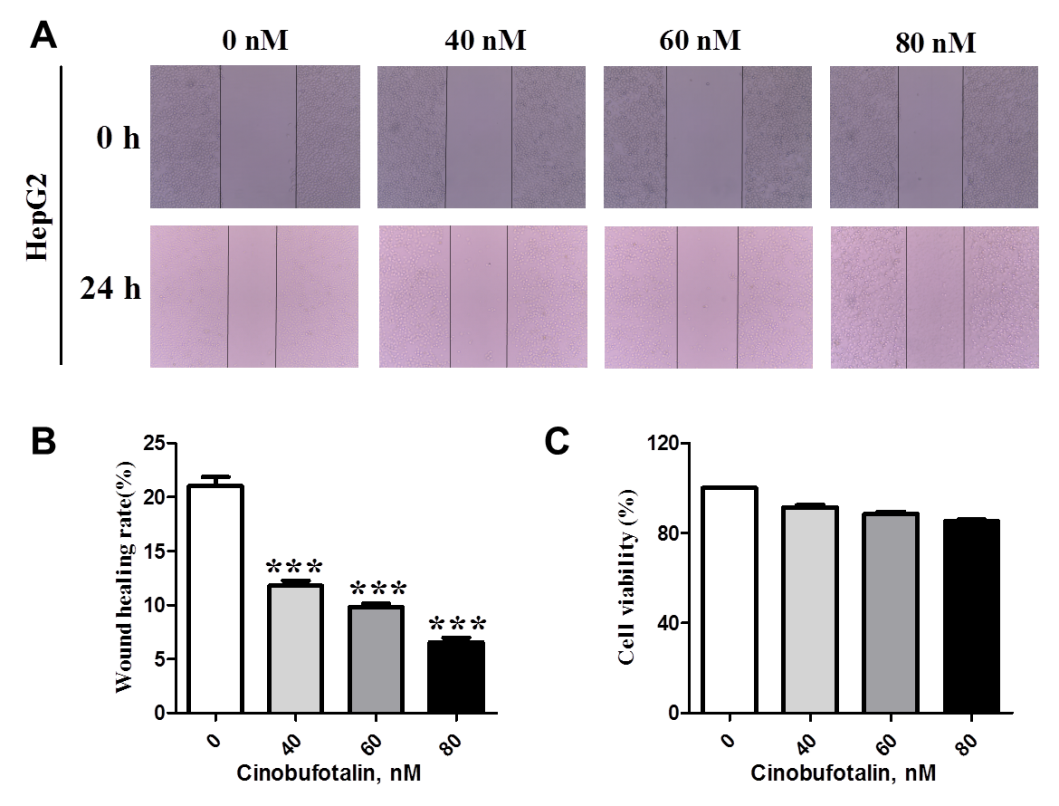


**Figure S2**


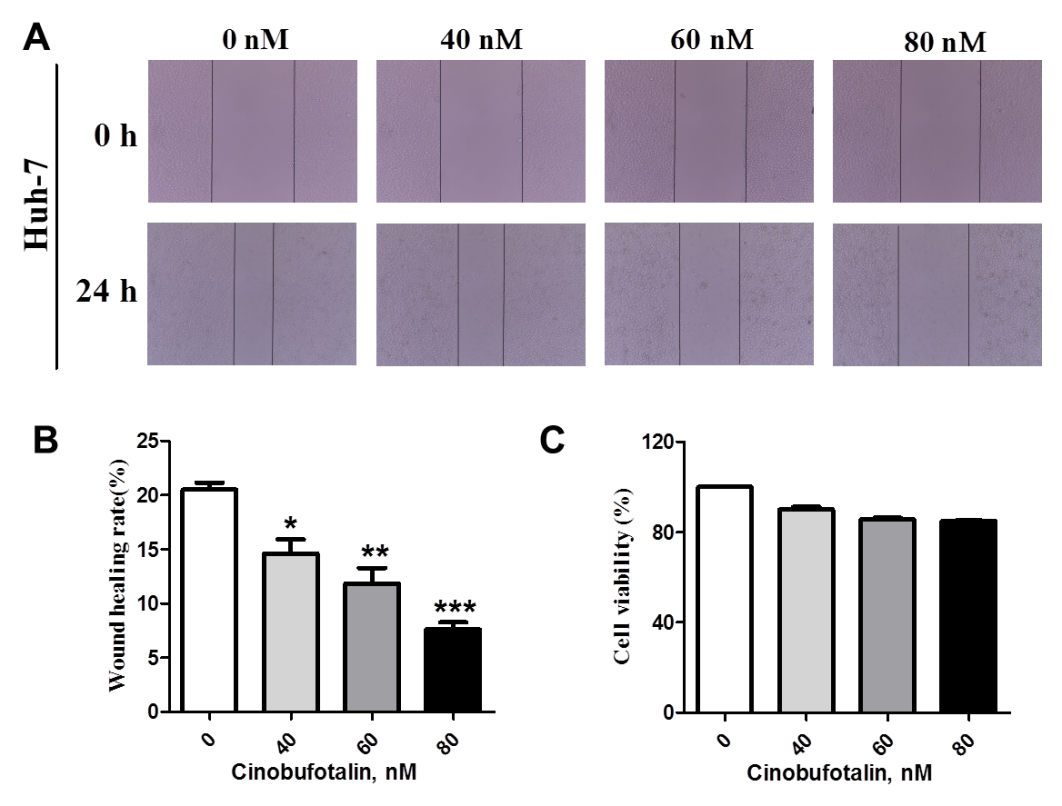

Supplement: Supplementary file 1 [file Data_Sheet_1.docx]
